# 4-Oxocyclo­hexa­neacetic acid: catemeric hydrogen bonding and spontaneous resolution of a single conformational enanti­omer in an achiral ∊-keto acid

**DOI:** 10.1107/S1600536810036652

**Published:** 2010-09-18

**Authors:** Alan Barcon, Andrew P. J. Brunskill, Roger A. Lalancette, Hugh W. Thompson

**Affiliations:** a Carl A. Olson Memorial Laboratories, Department of Chemistry, Rutgers University, Newark, NJ 07102, USA

## Abstract

The asymmetric unit of the title compound, C_8_H_12_O_3_, consists of a single conformational enanti­omer, which aggregates in the catemeric acid-to-ketone hydrogen-bonding mode [O⋯O = 2.682 (4) Å and O—H⋯O = 172 (6)°]. Four hydrogen-bonding chains of translationally related mol­ecules pass through the cell orthogonal to the 4_3_ screw axis along *c*, alternating in the 110 and the 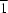
10 direction, with alignment with respect to this axis of + + − −. Successive chains are rotated by 90° around the *c* axis. One C—H⋯O=C close contact, involving the carboxyl group, exists.

## Related literature

For a discussion of highly ordered carboxyl bond distances and angles, see: Borthwick (1980 ▶). For close contact information, see: Steiner (1997 ▶). For related structures, see: Abell *et al.* (1991 ▶); Chen *et al.* (2000 ▶); Desiraju (1989 ▶); Halfpenny (1990 ▶); Jacques *et al.* (1981 ▶); Kawai *et al.* (1985 ▶); McGuire *et al.* (1995 ▶). For background information regarding the crystallization of a single chiral conformer from a racemic solution, see: Kondepudi *et al.* (1990 ▶). For *anti*-isoketopinic acid, see: Lalancette *et al.* (1997 ▶). For a description of the Cambridge Structural Database, see: Allen (2002 ▶).
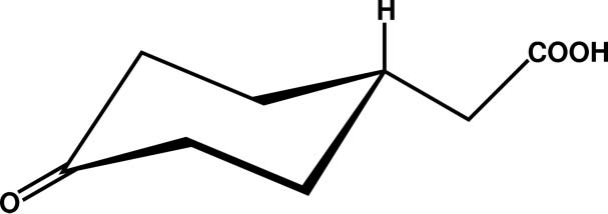


## Experimental

### 

#### Crystal data

C_8_H_12_O_3_
*M* *_r_* = 156.18
Tetragonal, 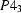

*a* = 6.8531 (12) Å
*c* = 17.372 (3) Å
*V* = 815.9 (3) Å^3^
*Z* = 4
Cu *K*α radiation
μ = 0.80 mm^−1^
*T* = 100 K
0.28 × 0.20 × 0.16 mm

#### Data collection

Bruker SMART APEXII CCD area-detector diffractometer
Absorption correction: multi-scan (*SADABS*; Sheldrick, 2008*a* ▶) *T*_min_ = 0.806, *T*_max_ = 0.882
6963 measured reflections
1310 independent reflections
1274 reflections with *I* > 2σ(*I*)
*R*_int_ = 0.029

#### Refinement

*R*[*F* ^2^ > 2σ(*F* ^2^)] = 0.039
*wR*(*F* ^2^) = 0.113
*S* = 1.18
1310 reflections
105 parameters
1 restraint
H atoms treated by a mixture of independent and constrained refinement
Δρ_max_ = 0.17 e Å^−3^
Δρ_min_ = −0.20 e Å^−3^
Absolute structure: Flack (1983 ▶), 559 Friedel pairs
Flack parameter: 0.0 (4)

### 

Data collection: *APEX2* (Bruker, 2006 ▶); cell refinement: *SAINT* (Bruker, 2005 ▶); data reduction: *SAINT*; program(s) used to solve structure: *SHELXTL* (Sheldrick, 2008*b* ▶); program(s) used to refine structure: *SHELXTL*; molecular graphics: *SHELXTL*; software used to prepare material for publication: *SHELXTL*.

## Supplementary Material

Crystal structure: contains datablocks I, global. DOI: 10.1107/S1600536810036652/sj5037sup1.cif

Structure factors: contains datablocks I. DOI: 10.1107/S1600536810036652/sj5037Isup2.hkl

Additional supplementary materials:  crystallographic information; 3D view; checkCIF report

## Figures and Tables

**Table 1:** Hydrogen-bond geometry (Å, °)

*D*—H⋯*A*	*D*—H	H⋯*A*	*D*⋯*A*	*D*—H⋯*A*
O3—H3⋯O1^i^	0.75 (6)	1.94 (6)	2.682 (4)	172 (6)
C7—H7*A*⋯O2^ii^	0.99	2.51	3.439 (5)	156

Symmetry codes: (i) 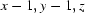; (ii) 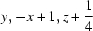.

**Fig. 1.**
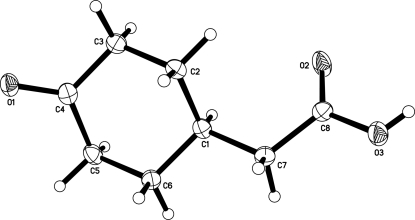
A view of the asymmetric unit with its numbering. Displacement ellipsoids are set at the 40% probability level.

**Fig. 2.**
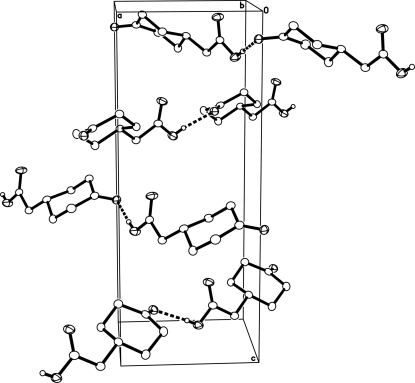
A packing diagram, with extracellular molecules, showing the four catemers created by acid-to-ketone H bonds proceeding along chains of translationally related molecules, advancing alternately along the 110 and the -110 directions. These chains have alignment with respect to the c axis of + + - -. All non-carboxyl H atoms have been removed for clarity. Displacement ellipsoids are set at the 30% probability level.

## supplementary crystallographic information

### Comment

The similar carbonyl basicities of carboxylic acids and ketones allow the two to compete as hydrogen-bond acceptors. Hence, simple keto acids display three known solid-state H-bonding modes beyond those seen in functionally unelaborated acids. The commonest, acid-to-ketone catemerization, constitutes a sizable minority of cases. The title compound aggregates as a catemer and crystallizes with only a single chiral conformer present.

Fig. 1 offers a view of the asymmetric unit. The expected staggering of substituents at C1 and C7 minimizes interactions with the axial H atoms at C2 and C6 by rotating the carboxyl away from the central O1—C4—C1—C7 plane, thus producing a chiral conformation. The C2—C1—C7—C8 torsion angle is -66.9 (4)° and the C1—C7—C8—O2 torsion angle is -9.9 (6)°.

The averaging of C—O bond lengths and C—C—O angles by disorder, common in carboxyl dimers, is not observed in catemers whose geometry cannot support the underlying averaging mechanisms involved. Here, these lengths and angles are typical of those for highly ordered dimeric carboxyls and catemers (Borthwick, 1980).

Fig. 2 shows the packing of the cell, with extracellular molecules to illustrate the H-bonding aggregation as translational carboxyl-to-ketone catemers around the 4_3_ screw axis coinciding with the c cell edge [O···O = 2.682 (4) Å, O—H···O = 172 (6)°]. Successive molecules in a given H-bonding chain advance alternately along the 110 and the -110 directions, with alignment with respect to the *c* axis of + + - -. Successive chains around the *c* axis are rotated by 90°. The structure therefore is comprised of stacked sheets in four orientations, each layer consisting of parallel H-bonding chains laid side-by-side. The arrangement is nearly identical with that we have previously reported for anti-isoketopinic acid (Lalancette *et al.*,  1997).

We characterize the geometry of H bonding to carbonyls using a combination of H···O=C angle and H···O=C—C torsion angle. These describe the approach of the acid H atom to the receptor O in terms of its deviation from, respectively, C=O axiality (ideal = 120°) and coplanarity with the carbonyl (ideal = 0°). Here, these two angles are 128.6 (16) and -1.2 (19)°.

Within the 2.6 Å range we survey for non-bonded C—H···O packing interactions (Steiner, 1997), only one close contact was found (see Table 2).

Although crystallization in space group P4_3_ is itself quite unusual (*ca* 0.12% of compounds in the Cambridge database), an equally unusual aspect of the packing is the presence of only a single chiral conformer. Preferential crystallization of one chiral conformer from solutions of an inherently achiral molecule is very rare but far from unknown (Jacques *et al.*, 1981; Desiraju, 1989). Among keto acids, five cases are known of this phenomenon: [Cambridge Structural Database (CSD, Version 5.28, update of Nov., 2006; Allen, 2002) refcodes CUHCUD (Kawai *et al.*, 1985), JISVAI (Abell *et al.*, 1991), KICRIX (Halfpenny, 1990) & ZEMJIK (McGuire *et al.*, 1995)], plus the case of mesitylglyoxylic acid (Chen *et al.*, 2000). The particular antipode crystallizing from such a solution may depend merely on which one chances to crystallize first, and it has been shown in a similar case that stirring seeds the solution and may largely or entirely prevent the enantiomeric species from crystallizing (Kondepudi *et al.*, 1990). In the present instance, the Flack parameter allows us to assign a specific hand to (I), so the antipode actually illustrated is the correct one. Also, the octant rule predicts that the conformer should have (-) rotation.

### Experimental

The solid-state (KBr) infrared spectrum of (I) has C=O stretching absorptions at 1726 and 1685 cm^-1^, consistent with known shifts produced when H-bonding is removed from carboxyl C=O and added to a ketone, respectively. In CHCl_3_ solution, these bands coalesce to a single absorption at 1707 cm^-1^, with a typical carboxyl-dilution shoulder around 1755 cm^-1^.

The title compound was prepared by Jones oxidation of the product obtained by catalytic hydrogenation of *p*-hydroxyphenylacetic acid over a Rh/C catalyst. The crystal used was obtained from Et_2_O/cyclohexane (60:40 v/v) by evaporation, mp 345 K.

### Refinement

All H atoms for (I) were found in electron density difference maps. The hydroxyl H was fully refined. The methylene and methine Hs were placed in geometrically idealized positions and constrained to ride on their parent C atoms with C—H distances of 0.99 and 1.00 Å, respectively, and *U*_iso_(H) = 1.2*U*_eq_(C).

### Crystal data

**Table:**

C_8_H_12_O_3_	*D*_x_ = 1.271 Mg m^−^^3^
*M**_r_* = 156.18	Melting point: 345 K
Tetragonal, *P*4_3_	Cu *K*α radiation, λ = 1.54178 Å
Hall symbol: P 4cw	Cell parameters from 6759 reflections
*a* = 6.8531 (12) Å	θ = 5.1–70.5°
*c* = 17.372 (3) Å	µ = 0.80 mm^−^^1^
*V* = 815.9 (3) Å^3^	*T* = 100 K
*Z* = 4	Block, colourless
*F*(000) = 336	0.28 × 0.20 × 0.16 mm

### Data collection

**Table:**

Bruker SMART APEXII CCD area-detector diffractometer	1310 independent reflections
Radiation source: fine-focus sealed tube	1274 reflections with *I* > 2σ(*I*)
graphite	*R*_int_ = 0.029
φ and ω scans	θ_max_ = 70.8°, θ_min_ = 6.5°
Absorption correction: multi-scan (*SADABS*; Sheldrick, 2008*a*)	*h* = −7→7
*T*_min_ = 0.806, *T*_max_ = 0.882	*k* = −8→7
6963 measured reflections	*l* = −20→18

### Refinement

**Table:**

Refinement on *F*^2^	Hydrogen site location: inferred from neighbouring sites
Least-squares matrix: full	H atoms treated by a mixture of independent and constrained refinement
*R*[*F*^2^ > 2σ(*F*^2^)] = 0.039	*w* = 1/[σ^2^(*F*_o_^2^) + (0.0369*P*)^2^ + 0.671*P*] where *P* = (*F*_o_^2^ + 2*F*_c_^2^)/3
*wR*(*F*^2^) = 0.113	(Δ/σ)_max_ < 0.001
*S* = 1.18	Δρ_max_ = 0.17 e Å^−^^3^
1310 reflections	Δρ_min_ = −0.20 e Å^−^^3^
105 parameters	Extinction correction: *SHELXTL* (Sheldrick, 2008b), Fc^*^=kFc[1+0.001xFc^2^λ^3^/sin(2θ)]^-1/4^
1 restraint	Extinction coefficient: 0.0023 (5)
Primary atom site location: structure-invariant direct methods	Absolute structure: Flack (1983), 559 Friedel pairs
Secondary atom site location: difference Fourier map	Flack parameter: 0.0 (4)

### Special details

**Table:**

Experimental. crystal mounted on a Cryoloop using Paratone-N
Geometry. All e.s.d.'s (except the e.s.d. in the dihedral angle between two l.s. planes) are estimated using the full covariance matrix. The cell e.s.d.'s are taken into account individually in the estimation of e.s.d.'s in distances, angles and torsion angles; correlations between e.s.d.'s in cell parameters are only used when they are defined by crystal symmetry. An approximate (isotropic) treatment of cell e.s.d.'s is used for estimating e.s.d.'s involving l.s. planes.
Refinement. Refinement of *F*^2^ against ALL reflections. The weighted *R*-factor *wR* and goodness of fit *S* are based on *F*^2^, conventional *R*-factors *R* are based on *F*, with *F* set to zero for negative *F*^2^. The threshold expression of *F*^2^ > σ(*F*^2^) is used only for calculating *R*-factors(gt) *etc*. and is not relevant to the choice of reflections for refinement. *R*-factors based on *F*^2^ are statistically about twice as large as those based on *F*, and *R*- factors based on ALL data will be even larger.

### Fractional atomic coordinates and isotropic or equivalent isotropic displacement parameters (Å^2^)

**Table:**

	*x*	*y*	*z*	*U*_iso_*/*U*_eq_	
O1	0.9958 (3)	1.2372 (3)	0.08526 (13)	0.0341 (5)	
O2	0.2472 (4)	0.6544 (3)	0.03744 (14)	0.0460 (6)	
O3	0.1245 (4)	0.5683 (4)	0.15016 (13)	0.0421 (6)	
H3	0.083 (6)	0.482 (6)	0.130 (2)	0.036 (11)*	
C1	0.4571 (4)	0.9768 (4)	0.10334 (18)	0.0275 (6)	
H1	0.3956	1.0155	0.0534	0.033*	
C2	0.6441 (4)	0.8617 (4)	0.08592 (17)	0.0289 (7)	
H2A	0.7056	0.8208	0.1349	0.035*	
H2B	0.6105	0.7426	0.0565	0.035*	
C3	0.7890 (4)	0.9841 (4)	0.03955 (19)	0.0299 (6)	
H3A	0.9128	0.9109	0.0342	0.036*	
H3B	0.7358	1.0064	−0.0127	0.036*	
C4	0.8297 (4)	1.1764 (4)	0.07670 (18)	0.0294 (7)	
C5	0.6532 (5)	1.2912 (4)	0.10375 (19)	0.0340 (7)	
H5A	0.5867	1.3494	0.0587	0.041*	
H5B	0.6967	1.3990	0.1376	0.041*	
C6	0.5086 (4)	1.1616 (4)	0.14778 (18)	0.0309 (7)	
H6A	0.5662	1.1249	0.1980	0.037*	
H6B	0.3879	1.2365	0.1580	0.037*	
C7	0.3105 (4)	0.8534 (4)	0.14873 (17)	0.0284 (6)	
H7A	0.3757	0.8031	0.1956	0.034*	
H7B	0.2019	0.9386	0.1656	0.034*	
C8	0.2272 (4)	0.6841 (4)	0.10499 (17)	0.0285 (7)	

### Atomic displacement parameters (Å^2^)

**Table:**

	*U*^11^	*U*^22^	*U*^33^	*U*^12^	*U*^13^	*U*^23^
O1	0.0344 (12)	0.0356 (12)	0.0322 (12)	−0.0107 (9)	−0.0004 (9)	0.0009 (9)
O2	0.0644 (16)	0.0488 (14)	0.0248 (12)	−0.0289 (12)	0.0023 (12)	−0.0052 (10)
O3	0.0544 (15)	0.0413 (14)	0.0305 (12)	−0.0195 (12)	0.0077 (11)	−0.0039 (11)
C1	0.0327 (15)	0.0281 (15)	0.0217 (14)	−0.0034 (12)	−0.0037 (12)	0.0028 (12)
C2	0.0366 (16)	0.0244 (15)	0.0258 (16)	−0.0003 (12)	−0.0009 (13)	−0.0002 (12)
C3	0.0321 (16)	0.0318 (15)	0.0257 (15)	0.0002 (12)	−0.0033 (13)	0.0002 (13)
C4	0.0353 (16)	0.0308 (15)	0.0222 (14)	−0.0054 (13)	−0.0014 (13)	0.0070 (12)
C5	0.0407 (18)	0.0254 (16)	0.0358 (18)	−0.0041 (13)	−0.0004 (14)	−0.0018 (13)
C6	0.0282 (15)	0.0294 (16)	0.0352 (17)	0.0007 (11)	−0.0004 (13)	−0.0025 (14)
C7	0.0311 (15)	0.0299 (15)	0.0244 (15)	−0.0004 (11)	−0.0004 (12)	0.0015 (12)
C8	0.0270 (15)	0.0351 (16)	0.0234 (16)	0.0001 (12)	−0.0016 (12)	0.0002 (12)

### Geometric parameters (Å, °)

**Table:**

O1—C4	1.222 (4)	C3—H3A	0.9900
O2—C8	1.199 (4)	C3—H3B	0.9900
O3—C8	1.319 (4)	C4—C5	1.518 (4)
O3—H3	0.74 (4)	C5—C6	1.535 (4)
C1—C6	1.524 (4)	C5—H5A	0.9900
C1—C7	1.532 (4)	C5—H5B	0.9900
C1—C2	1.535 (4)	C6—H6A	0.9900
C1—H1	1.0000	C6—H6B	0.9900
C2—C3	1.529 (4)	C7—C8	1.500 (4)
C2—H2A	0.9900	C7—H7A	0.9900
C2—H2B	0.9900	C7—H7B	0.9900
C3—C4	1.494 (4)		
			
C8—O3—H3	114 (3)	C4—C5—C6	111.6 (2)
C6—C1—C7	110.5 (2)	C4—C5—H5A	109.3
C6—C1—C2	109.5 (2)	C6—C5—H5A	109.3
C7—C1—C2	111.4 (2)	C4—C5—H5B	109.3
C6—C1—H1	108.5	C6—C5—H5B	109.3
C7—C1—H1	108.5	H5A—C5—H5B	108.0
C2—C1—H1	108.5	C1—C6—C5	112.2 (2)
C3—C2—C1	111.4 (2)	C1—C6—H6A	109.2
C3—C2—H2A	109.4	C5—C6—H6A	109.2
C1—C2—H2A	109.4	C1—C6—H6B	109.2
C3—C2—H2B	109.4	C5—C6—H6B	109.2
C1—C2—H2B	109.4	H6A—C6—H6B	107.9
H2A—C2—H2B	108.0	C8—C7—C1	114.6 (2)
C4—C3—C2	112.2 (3)	C8—C7—H7A	108.6
C4—C3—H3A	109.2	C1—C7—H7A	108.6
C2—C3—H3A	109.2	C8—C7—H7B	108.6
C4—C3—H3B	109.2	C1—C7—H7B	108.6
C2—C3—H3B	109.2	H7A—C7—H7B	107.6
H3A—C3—H3B	107.9	O2—C8—O3	122.8 (3)
O1—C4—C3	121.8 (3)	O2—C8—C7	125.7 (3)
O1—C4—C5	121.9 (3)	O3—C8—C7	111.5 (3)
C3—C4—C5	116.3 (3)		
			
C6—C1—C2—C3	−58.4 (3)	C7—C1—C6—C5	−179.2 (2)
C7—C1—C2—C3	179.1 (2)	C2—C1—C6—C5	57.8 (3)
C1—C2—C3—C4	52.9 (3)	C4—C5—C6—C1	−51.0 (3)
C2—C3—C4—O1	132.3 (3)	C6—C1—C7—C8	171.1 (2)
C2—C3—C4—C5	−47.3 (4)	C2—C1—C7—C8	−67.0 (3)
O1—C4—C5—C6	−133.5 (3)	C1—C7—C8—O2	−9.6 (4)
C3—C4—C5—C6	46.0 (4)	C1—C7—C8—O3	171.0 (3)

### Hydrogen-bond geometry (Å, °)

**Table:**

*D*—H···*A*	*D*—H	H···*A*	*D*···*A*	*D*—H···*A*
O3—H3···O1^i^	0.75 (6)	1.94 (6)	2.682 (4)	172 (6)
C7—H7A···O2^ii^	0.99	2.51	3.439 (5)	156

Symmetry codes: (i) *x*−1, *y*−1, *z*; (ii) *y*, −*x*+1, *z*+1/4.
